# Analysis of Spatial Organization of Suppressive Myeloid Cells and Effector T Cells in Colorectal Cancer—A Potential Tool for Discovering Prognostic Biomarkers in Clinical Research

**DOI:** 10.3389/fimmu.2020.550250

**Published:** 2020-10-29

**Authors:** Natalie Zwing, Henrik Failmezger, Chia-Huey Ooi, Derrek P. Hibar, Marta Cañamero, Bruno Gomes, Fabien Gaire, Konstanty Korski

**Affiliations:** ^1^ Early Biomarker Development Oncology, pharma Research and Early Development (pRED), Roche Innovation Center Munich, Penzberg, Germany; ^2^ pharma Research and Early Development Informatics (pREDi), Roche Innovation Center Munich, Penzberg, Germany; ^3^ Pharmaceutical Sciences—Biomarkers, Bioinformatics and Omics (PS-BiOmics), pharma Research and Early Development (pRED), Roche Innovation Center Basel, Basel, Switzerland; ^4^ Product Development, Personalized Healthcare Analytics, Genentech, Inc., South San Francisco, CA, United States; ^5^ Early Biomarker Development Oncology, pharma Research and Early Development (pRED), Roche Innovation Center Basel, Basel, Switzerland; ^6^ Product Development, Personalized Healthcare Data Science Imaging, Roche Pharma, Basel, Switzerland

**Keywords:** computational pathology, spatial statistics, tumor immune microenvironment, suppressive myeloid cells, T cells, colorectal cancer ****

## Abstract

The development and progression of solid tumors such as colorectal cancer (CRC) are known to be affected by the immune system and cell types such as T cells, natural killer (NK) cells, and natural killer T (NKT) cells are emerging as interesting targets for immunotherapy and clinical biomarker research. In addition, CD3^+^ and CD8^+^ T cell distribution in tumors has shown positive prognostic value in stage I–III CRC. Recent developments in digital computational pathology support not only classical cell density based tumor characterization, but also a more comprehensive analysis of the spatial cell organization in the tumor immune microenvironment (TiME). Leveraging that methodology in the current study, we tried to address the question of how the distribution of myeloid derived suppressor cells in TiME of primary CRC affects the function and location of cytotoxic T cells. We applied multicolored immunohistochemistry to identify monocytic (CD11b^+^CD14^+^) and granulocytic (CD11b^+^CD15^+^) myeloid cell populations together with proliferating and non-proliferating cytotoxic T cells (CD8^+^Ki67^+/–^). Through automated object detection and image registration using HALO software (IndicaLabs), we applied dedicated spatial statistics to measure the extent of overlap between the areas occupied by myeloid and T cells. With this approach, we observed distinct spatial organizational patterns of immune cells in tumors obtained from 74 treatment-naive CRC patients. Detailed analysis of inter-cell distances and myeloid-T cell spatial overlap combined with integrated gene expression data allowed to stratify patients irrespective of their mismatch repair (MMR) status or consensus molecular subgroups (CMS) classification. In addition, generation of cell distance-derived gene signatures and their mapping to the TCGA data set revealed associations between spatial immune cell distribution in TiME and certain subsets of CD8^+^ and CD4^+^ T cells. The presented study sheds a new light on myeloid and T cell interactions in TiME in CRC patients. Our results show that CRC tumors present distinct distribution patterns of not only T effector cells but also tumor resident myeloid cells, thus stressing the necessity of more comprehensive characterization of TiME in order to better predict cancer prognosis. This research emphasizes the importance of a multimodal approach by combining computational pathology with its detailed spatial statistics and gene expression profiling. Finally, our study presents a novel approach to cancer patients’ characterization that can potentially be used to develop new immunotherapy strategies, not based on classical biomarkers related to CRC biology.

## Introduction

Currently used classification of colorectal cancer (CRC) tumors is based on classical pathological features such as tumor architecture, infiltration of bowel wall, and involvement of local lymph nodes assessed in the HE stained slides. Despite being clinically relevant, pathology staging shows its limitations especially in the era of cancer immunotherapy (CIT). With pembrolizumab being registered for Microsatellite Instable (MSI) tumors irrespective of the cancer type (1, 2), the role of non-classical parameters like Mismatch Repair (MMR) status, tumor infiltrating lymphocyte (TIL) density, or tumor mutation burden (TMB) in predicting patients outcome increased dramatically (3, 4). However, since many of the CIT regiments target directly T effector cells (5, 6), ongoing research tries to address primarily T cell biology partially overlooking the importance of other immune cell types in shaping the tumor immune microenvironment (TiME). One of them, namely tumor myeloid derived suppressor cells (MDSC), has been postulated to play an important role in generating a suppressive environment negatively affecting the function of T effector cells (7, 8). In the present study, we address the question of how the distribution of certain types of MDSC in TiME of primary CRC impact the location and function of cytotoxic T cells. By leveraging computational pathology and spatial statistics (9), we identified distinct spatial organizational patterns of immune cells in the CRC TiME. Our research suggests that the proximity of monocytic and granulocytic myeloid and cytotoxic T cells may reflect their functional interactions. The spatial analysis indicates that the location of monocytic cells correlates with the presence of TCF7 memory stem-like lymphocytes and tumor specific T cells, whereas spatial distribution of granulocytic cells associates with the activity of CD4^+^ lymphocytes. In addition, our approach allowed to stratify CRC patients into 4 categories according to the level of overlap between myeloid and T cells irrespective of the MMR and CMS status. Categories with high levels of spatial overlap generally revealed down-regulation of cytotoxic T cell related pathways.

## Materials and Methods

### CRC Samples

Human primary CRC tumor specimens of 74 treatment-naïve patients were acquired from Avaden Biosciences and Indivumed. The samples were collected after obtaining patients informed consent and approval from the respective Institutional Review Boards or equivalent agencies. For all patients, additional clinical information was provided, including gender, age, tumor stage and grade, tumor-node-metastasis (TNM) classification, tissue of excision detail, MMR status, CMS classification and TMB Score (**Table 1**). Fresh specimens were prepared as formalin fixed and paraffin embedded tissue (FFPET) blocks prior to shipment and further used for either chromogenic immunohistochemistry (IHC) staining or RNA extraction. The applied tissue processing workflow is presented in the Supplementary Material (**Supplementary Figure S3**), which includes all steps described in the following paragraphs.

**Table 1:** Clinical Data of CRC patients used in this study.

Parameters	No. of patients	percent (%)
**Gender**		
Male	36	49
Female	38	51
**Age**		
< 50	3	4
50–70	14	19
≥ 70	57	77
**Tumor Excision**		
Right-sided	13	18
Left-sided	16	22
Rectum	17	23
NA	28	39
**Tumor Grade**		
Grade 1	8	11
Grade 2	42	57
Grade 3	24	32
**Tumor Stage**		
Stage I	2	3
Stage II	10	14
Stage III	15	20
Stage IV	47	64
**pTNM Status**		
pT1	0	0
pT2	5	7
pT3	49	66
pT4	20	27
pN0	19	26
pN1	23	31
pN2	32	43
pMX	26	35
pM0	1	1
pM1	47	64
**MMR Status**		
MSI	17	23
MSS	57	77

### IHC Staining Protocols

Sections of 2.5 *μm* thickness were stained with following single- and double colored chromogenic immune assays: CD11b/CD14, CD11b/CD15, CD8/Ki67, ARG1, and FOXP3. Staining procedures were performed, using Ventana Discovery Ultra, Discovery XT, or Benchmark XT automated stainers (Ventana Medical Systems, Tucson, AZ) with NEXES version 10.6 software. For all IHC assays, sections were first dewaxed, antigens were retrieved with Tris-EDTA based Cell Conditioning 1 and peroxidase inhibitor was applied to decrease endogenous peroxidase activity. For the myeloid duplex assays, CD11b/CD14 and CD11b/CD15, the primary antibody CD11b (Abcam, EPR1344, 1:400) was applied for 32 min at 37°C and then detected with UltraMap anti-rabbit HRP secondary antibody and subsequent Discovery Purple detection kit (Ventana Medical Systems). After heat denaturation, second primary antibody, either CD14 (Ventana Medical Systems, EPR3635, RTU) or CD15 (Ventana Medical Systems, MMA, RTU), was applied for 32 min at 37°C and detected with either UltraMap anti-rabbit AP or UltraMap anti-mouse AP secondary antibody and subsequent Discovery Yellow detection kit (Ventana Medical Systems). Sections stained with CD8/Ki67 assay were first incubated with primary antibody CD8 (Spring Biosciences, SP239, 1:12.5) for 32 min at 37°C. Bound CD8 antibody was detected with UltraMap anti-rabbit AP secondary antibody and Discovery Yellow detection kit (Ventana Medical Systems). The second primary antibody Ki67 (Ventana Medical Systems, 30-9, RTU) was added after heat denaturation for 8 min at 37°C, then detected with Hapten linked Multimer anti-rabbit HQ and anti-HQ HRP secondary antibody, followed by Discovery Purple detection kit (Ventana Medical Systems). For ARG1 assay, sections were first treated with primary antibody ARG1 [Abcam, EPR6672(B), 1:500] for 60 min at 37°C and bound antibody was detected with OmniMap anti-rabbit HRP secondary antibody and ChromoMap DAB detection kit (Ventana Medical Systems). As last, sections stained with FOXP3 assay were incubated with primary antibody FOXP3 (Abcam, 236A-E7, 1:100) for 60 min at 37°C and positive staining was detected with OptiView DAB detection kit (Ventana MedicalSystems). The nuclear counterstaining was implied for all assays by adding both Hematoxylin II and Bluing Reagent for 8 min each. Finally, slides were dehydrated and coverslipped with a permanent mounting medium.

### Digital Image Analysis

Immunostained slides were histologically evaluated by an expert pathologist and then digitally scanned at 20X magnification with the high throughput iScan HT (Ventana Medical Systems). Whole-slide images were analyzed with the HALO Software (IndicaLabs) tool. On each image, tumor and normal colon regions were manually annotated and substantial areas of necrosis or tissue artefacts were excluded. The invasive margin was automatically applied, with a 500 *µm* width, encompassing both tumor and normal colon regions at 250 *µm* each. Images of the slides stained with CD8/Ki67 were registered to the images of consecutively cut slides of CD11b/CD14 and CD11b/CD15 to transfer annotations and for further spatial analysis. Annotations of ARG1 and FOXP3 images were processed separately. Next, images were used for training the algorithms to detect monocytic CD11b^+^CD14^+^ and granulocytic CD11b^+^CD15^+^ myeloid cells, ARG1^+^ immunosuppressive myeloid cells, proliferating and non-proliferating CD8^+^Ki67^+/–^ cytotoxic and regulatory FOXP3^+^ T cells (**Figure 2A**). Total cell counts, annotation areas and cell object XY coordinates were extracted for tumor, invasive margin and normal colon regions of interest (ROI). Spatial maps combining Ki67^+^ tumor cells, CD8^+^Ki67^+/–^ T cells and either CD11b^+^CD14^+^ or CD11b^+^CD15^+^ myeloid cells, were used to visualize distribution patterns of immune cells in tumor annotated areas and to further perform spatial overlap analysis (see chapter 2.4.2). To provide additional information about cell co-localization at higher resolution, distances between myeloid cells and T cells were measured. Briefly, each detected CD8^+^ cell was assigned to the nearest respective myeloid cell, the distances between formed cell pairs were extracted and a global average distance (GAD) per CRC sample was extracted.

### Spatial Analysis

#### GAD*_norm_*parameter

To avoid potential bias from the amount of myeloid cells, the GAD was normalized against the myeloid cell density and the expected mean distance for a random distribution pattern of myeloid cells (10):

(1) $$GAD_{norm}=\frac{GAD}{\frac{0.5}{\sqrt{\frac{n}{A}}}}$$

Here (1), *n* corresponds to the total number of myeloid cells (CD11b^+^CD14^+^ or CD11b^+^CD15^+^) and *A* to the annotated tumor area. To differentiate between (CD11b^+^CD14^+^ and (CD11b^+^CD15^+^ derived GAD*_norm_*parameters we called them: GAD_CD14 and GAD_CD15, respectively. These parameters were then used for further gene correlation analysis (see chapter 2.6).

#### Spatial Overlap Analysis

For better understanding the spatial relation between myeloid and T cells in the TiME, we calculated the spatial overlap between (CD11b^+^CD14^+^ or CD11b^+^CD15^+^) myeloid cells and CD8^+^Ki67^+/–^ T cells. First, spatial maps of annotated tumor regions, including tumor cell, myeloid cell and T cell XY coordinates, were overlaid with a hexagonal grid displayed with a diagonal length of 250 *µm* (**Figure 4A**). For each single tile, we computed cell densities of both myeloid and T cells, which were then compared with corresponding median tumor density of the whole CRC cohort. Tiles with immune cell densities measured above the median tumor density were labelled with “hot” for respective CD8^+^, CD11b^+^CD14^+^, and CD11b^+^CD15^+^ (2). So, we defined a tile *h* with index *i* to be “hot” for a cell type *j* if:

(2) $$h_{ij}=\{\begin{array}{l} 1\;d_{ij}>D_{j}\\ 0\;otherwise \end{array}$$

with *I* = 1*,…, N*, whereas *d_ij_* representing the density of a cell type *j* in tile *i* and *D_j_* the median cell density.

This process resulted in a tiled spatial distribution map, representing single T cell or myeloid cell “hot” tiles and additional overlapping “hot” tiles with both high T cell and myeloid cell density. In order to determine the amount of CD8^+^ “hot” areas that were also occupied by myeloid cells (3), we counted overlap tiles that were both CD8^+^ and CD11b^+^CD14^+^ or CD11b^+^CD15^+^ “hot” and normalized this value by the total number of CD8^+^ “hot” tiles per sample. The Myeloid-T cell Overlap (MTO) parameter for two cell types *j,k* was calculated as in the following equation:

(3) $$MTO_{j,k}=\frac{\Sigma_{i=1}^{N}\;h_{ij}*h_{ik}}{\Sigma_{i=1}^{N}\;h_{ij}}$$

Here (3), *j* accounted for cytotoxic CD8^+^ T cells and *k* either for CD11b^+^CD14^+^ or CD11b^+^CD15^+^ myeloid cells. To differ between MTO calculated for CD11b^+^CD14^+^ myeloid cells and MTO calculated for CD11b^+^CD15^+^ myeloid cells, we named the respective parameters MTO_CD14 and MTO_CD15. The MTO parameters were further plotted against tumor CD8^+^ T cell density in order to better characterize the CRC cases according to the different levels of T cell infiltration Using their median values of both parameters (CD8^+^ T cell density/MTO level), we stratified the CRC patients into four categories: low/low (category 1), low/high (category 2), high/high (category 3), and high/low(category 4).

### RNA Extraction and Sequencing

The AllPrep DNA/RNA FFPE Kit (Qiagen Cat No./ID: 80234) was used to purify genomic DNA and total RNA from 10 *µm* thick FFPET curls, according to the manufacturer’s instructions. RNA samples were then assessed for quality and quantity using the Qubit instrument and the Agilent Bioanalyzer to determine the degradation of the RNA samples (DV200 value). To further generate the sequencing library, the hybridization-based Illumina TruSeq RNA Access method was performed according to the manufacturer’s instructions, with first preparation of the total RNA library and second library enrichment for coding RNA. Finally, normalized libraries were sequenced using the Illumina sequencing-by-synthesis platform, with a sequencing protocol of 50 bp paired-end sequencing and total read depth of 25M reads per sample.

### Gene Expression and Correlative Analysis

#### Correlation Analysis With Distance Parameter GAD*_norm_*

A single sample signature scoring method, BioQC (11) was adapted to compute signature scores for patient samples in both our CRC cohort and TCGA. We performed a centering and rescaling transformation on the rank-biserial correlation output by BioQC. First, rank-biserial correlation values were multiplied by 10, and then median-centered for each signature. This is to enable qualitative comparison to gene expression values (log2 RPKM) and comparison across samples (a score above 0 indicates the sample is enriched in the signature compared to at least half of the population in the CRC cohort or TCGA dataset).

Spearmans rank correlation coefficient was then used to quantify the strength and direction of the association between a signature or gene and either the 1) measured GAD*_norm_*parameter in the CRC cohort, or 2) signature representing the GADrscore CD14 or GAD_CD15 in the TCGA dataset, respectively. To identify the signature in point 2), we used the following cutoff for GAD_CD14 based correlation (|*R*| *>* 0.4): 64 genes in total (32 positively correlated genes, 32 negatively correlated genes) and for GAD_CD15 based correlation (|*R*| *>* 0.3): 271 genes in total (180 positively correlated genes, 91 negatively correlated genes) (**Supplementary Table S2**).

The permutation test was performed to evaluate the correlation between a signature or gene of interest and the signature representing the global average distance (GAD_CD14 or GAD_CD15) (“distance signature” for short). The steps in the permutation test for a signature or gene of interest (SGOI) were as follows: first we computed the correlation coefficients of the distance signature to the SGOI in each cancer cohort. Next, we generated 10,000 random signatures of similar size to the distance signature and we computed the correlation coefficients for each random signature to the SGOI in each cancer cohort. We counted how many times a random signature had absolute correlation coefficient that exceeds the absolute correlation from the distance signature. Finally, we divided this number by the total number of random signatures in order to get the p-value for the null hypothesis that the correlation of the SGOI to the distance signature could have occurred by random chance alone.

#### Differential Gene Expression and Pathway Analysis With Spatial Overlap Categories

redFor this analysis we used the DESeq2 (12) analysis pipeline to investigate group differences in gene expression derived from RNAseq summarized gene count data. More specifically, we compared differences in gene expression between spatial overlap category 1 and 2 and separately between category 3 and 4 in order to understand genetic differences associated with high vs low overlap when CD8^+^ T cell density is high vs low. We made these comparisons for each of the myeloid cell subtypes of interest (i.e. (CD11b^+^CD14^+^ and CD11b^+^CD15^+^). For all comparisons, the high vs low group label was assigned using a median cutoff. We further investigated whether genes associated with differences in Myeloid-T cell Overlap were associated with dysregulation of specific pathways using the LRpath analysis pipeline (13) using the RNA-enrich option to account for biases associated with gene count.

### Statistical Methods

All statistical analysis was carried out using R (14). Statistical significance for difference between IHC extracted features were assessed with Mann-Whitney U Test. For correlation analysis between cell densities in tumor ROI, the Pearson Correlation Coefficients were calculated. The R package spatstat was used for spatial analysis (15) and GGplot2 was used for visualization (16).

## Results

### T Cell and Myeloid Cell Subpopulations Are Distinctly Distributed Across Tumor Stromal and Tumor Epithelial Compartments

**Figure 1:**
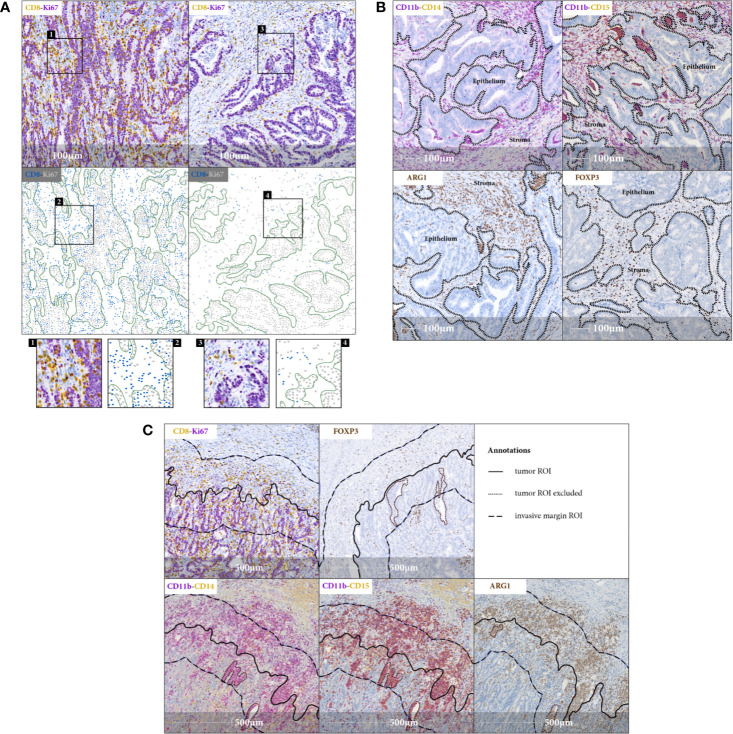
Immune cell distribution in the TiME. **(A)** Representative IHC images (20x magnification) and corresponding spatial maps for CD8^+^ cytotoxic T cell distribution in tumor stromal (right panel) and tumor epithelial (left panel) compartments indicating variety of lymphocytic infiltration patterns observed in CRC samples. Involvement of tumor epithelium by CD8^+^ T cells depicted in the left panel representing an exemplary MSI case. CD8^+^Ki67^+^ staining serves as a surrogate marker for proliferating cancer cells. Green lines in spatial maps are manually annotated for FOVs and mark the tumor epithelial border for better visualization. Detailed view in the inlets. **(B)** FOVs, showing tumor stromal immune infiltration of CD11b^+^CD14^+^, CD11b^+^CD15^+^ and ARG1^+^ myeloid cells and FOXP3^+^ regulatory T cells, with black dashed lines indicating the epithelial borders. There is a striking accumulation of myeloid cells predominantly in tumor stromal compartment **(C)** IHC images (20x magnification), representing myeloid and T cell distribution in invasive margin ROI. Note the aggregation of myeloid cells along the tumor invasive front.

The observation of digital images representing sections stained with myeloid and T cell markers, shows specific immune cell distribution patterns in the TiME. In the tumor ROI, we focused on stromal and epithelial tumor compartments and assessed their infiltration by monocytic CD11b^+^CD14^+^, granulocytic CD11b^+^CD15^+^, and immunosuppressive ARG1^+^ myeloid cells and also CD8^+^Ki67^+/–^ cytotoxic and FOXP3^+^ regulatory T cells. Cytotoxic T cell infiltration is observed in most cases only in the stromal compartment. Minority of cases, mostly MSI type, show additional CD8^+^ T cell infiltration into the epithelial tumor compartment (**Figure 1A**). On the contrary, myeloid cells and regulatory T cells exclusively occupy the stromal compartment, as their distribution pattern in the tumor ROI is mirroring the distribution of the stroma itself (**Figure 1B**). In the invasive margin ROI, both myeloid and T cells tend to accumulate, with myeloid infiltration being relatively higher. CD11b^+^ and ARG1^+^ myeloid cells are co-localizing close to the tumor epithelial border, forming an envelope covering the invasive front of the tumor ROI (**Figure 1C**).

### Myeloid Cell Populations Show the Highest Density in the Invasive Margin ROI and Have Significantly Higher Density in Tumor ROI of MSI Cases

**Figure 2:**
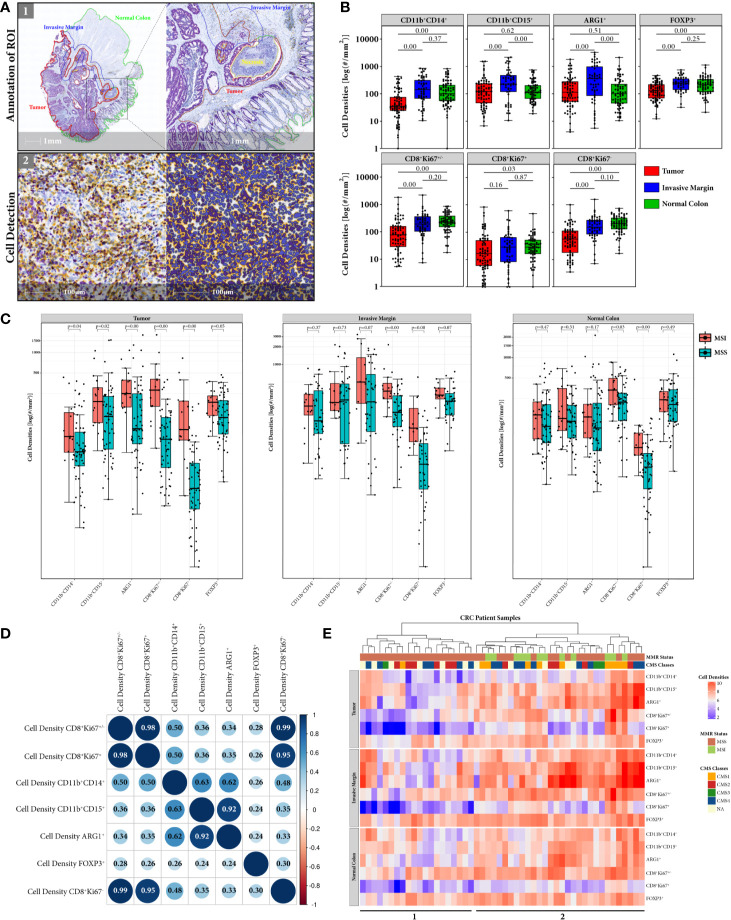
Immune cell densities computed in tumor, invasive margin and normal colon ROIs. **(A)** HALO Workflow for processing immune cell detection in all annotated respective ROIs. **(B)** Global distribution of myeloid and T cell densities for tumor (N = 74), invasive margin (N = 51), and normal colon (N = 69) ROI. Distribution of cell densities across the compartments suggests that despite the accumulation of immune cells in tumor invasive front the tumor bed is dominated by myeloid and T regulatory immunosuppressive populations. **(C)** Comparison of myeloid and T cell densities according to CRC patients MMR status (MSI = red, MSS = blue) in all 3 ROIs. Significant difference between MSS and MSI cases are observed for immunosuppresive cells only in tumor ROI **(D)** Correlation matrix of myeloid and T cells densities measured in annotated tumor ROI. Sizes of the circles correspond to the strength of the Pearson correlation coefficient (inside the circles) and colors correspond to the direction of the correlation (blue for positive, red for negative). Note strong correlation between ARG1^+^ and CD11b^+^CD15^+^ cell densities indicating the immunosuppressive nature of granulocytic myeloid cells. Weak to moderate correlation between densities of cytotoxic T cells and myeloid cell subpopulations suggests existence of other contributing factors like the spatial distribution. **(E)** Heatmap representation of hierarchical clustering of CRC patients (columns) according to their measured immune cell densities (rows) in tissue annotated regions. Only patient specimens having all 3 ROIs (N = 50) were used for the cluster analysis. Color coded bars corresponding to the MMR status and CMS classification of the cases were added on the top of the heatmap to illustrate the distribution of MMR and CMS categories across clustered samples. Cluster 1 represents generally lower myeloid cell content and consists exclusively of MSS cases, while cluster 2 shows generally higher immune cell infiltration in both MSS and MSI cases.

Single immune cells were detected with trained algorithms in annotated tumor, invasive margin and normal colon ROI and cell densities of CD11b^+^CD14^+,^ CD11b^+^CD15^+^ and ARG1^+^ myeloid cells, and CD8^+^Ki67^+/–^, CD8^+^Ki67^+/–^ and FOXP3^+^ T cells were computed respectively (**Figures 2A, B**). Generally, the invasive margin ROI shows the highest immune cell infiltration among all three analyzed ROIs with median cell densities: 142.82 and 227.42 cells/mm^2^ for monocytic and granulocytic myeloid cells, respectively, 192.01 and 244.08 cells/mm^2^ for cytotoxic and regulatory T cells, respectively. Interestingly, the measured cell densities are significantly higher than in the tumor ROI, which is dominated by immunosuppressive myeloid and regulatory T cells with median densities: 35.32 and 122.12 cells/mm^2^ formonocytic and granulocytic myeloid cells, respectively, 76.64 and 129.89 cells/mm^2^ for cytotoxic and regulatory T cells, respectively. On the contrary, in the normal colon ROI both T cell subpopulations represent higher median cell densities than myeloid cells: 102.29 and 116.19 cells/mm^2^ for monocytic and granulocytic myeloid cells, respectively, 233.32 and 214.11 cells/mm^2^ for cytotoxic and regulatory T cells, respectively. Despite the fact that cytotoxic CD8^+^Ki67^+/–^ T cells show the lowest density in tumor ROI, the proportion of proliferating CD8^+^Ki67^+/–^ T cells to total CD8^+^Ki67^+/–^ T cells is higher than in invasive margin and normal colon ROIs (**Supplementary Figure S4**).

Comparing the myeloid cell subpopulations, the monocytic cells exhibit the lowest median density values, in both tumor and invasive margin ROI. The immune cell population expressing the suppressive ARG1^+^ marker, follows the granulocytic myeloid cell expression levels in all ROIs. This is also reflected by strong positive correlation (Pearson 0.92) between ARG1^+^ and CD11b^+^CD15^+^ myeloid cell densities in tumor ROI (**Figure 2D**). The monocytic myeloid cell density, on the contrary, shows only a moderate positive correlation to ARG1^+^ cell density (Pearson 0.62). In addition, redproliferating CD8^+^Ki67^+/–,^non-proliferating CD8^+^Ki67^+/–^ and total CD8^+^Ki67^+/–^ cytotoxic T cells show a moderate positive correlation with monocytic cell density (Pearson 0.50, 0.48, and 0.50, respectively) and a weak positive correlation with granulocytic myeloid cell density (Pearson 0.36, 0.35, and 0.36, respectively). T regulatory cell density is very weakly positively correlated with the densities of other studied immune cell types. The same correlation analysis was performed in the invasive margin ROI (**Supplementary Figure S5**), only showing weak correlations between monocytic myeloid cells and proliferating, non-proliferating and total cytotoxic T cells (Pearson 0.18, 0.09, and 0.12, respectively) and weak correlations between granulocytic myeloid cells and corresponding cytotoxic T cells (Pearson 0.10, 0.14, and 0.13, respectively).

When comparing cell densities according to the patients MMR status, we observe a generally higher immune cell density in MSI cases in all ROIs, for both myeloid and T cells (**Figure 2C**). However, myeloid and T regulatory cells show significant difference only in tumor ROI, whereas proliferating and non-proliferating cytotoxic CD8^+^ T cells are significantly higher in MSI throughout all tissue ROIs.

In addition, when cell densities of each immune cell type in all three ROIs were analyzed through hierarchical clustering, CRC patients with higher myeloid content (cluster 2) tend to cluster with both CD8^+^ T cell high and low density groups (**Figure 2E**). Interestingly, this group of patients represents both MSI (N = 12) and MSS (N = 18) cases, whereas the cluster with low CD8^+^ infiltration and lower myeloid content (cluster 1) only includes MSS patients (N = 20). The CMS classes, however, are inconsistently distributed throughout cluster 1 and 2, only CMS1 follows the pattern of MSI patients with one exceptional CMS1 case in cluster 1. The T regulatory cell densities were equally distributed throughout both patient clusters.

### The Average Distance Between Monocytic Myeloid Cells and Cytotoxic T Cells Is Higher in MSI Cases

In order to characterize further distribution of myeloid and T cells and their spatial relation, we used the normalized distance parameter GAD*_norm_* of CD8^+^ T cells to CD11b^+^CD14^+^ and CD11b^+^CD15^+^ (GAD_CD14 and GAD_CD15), respectively.

**Figure 3:**
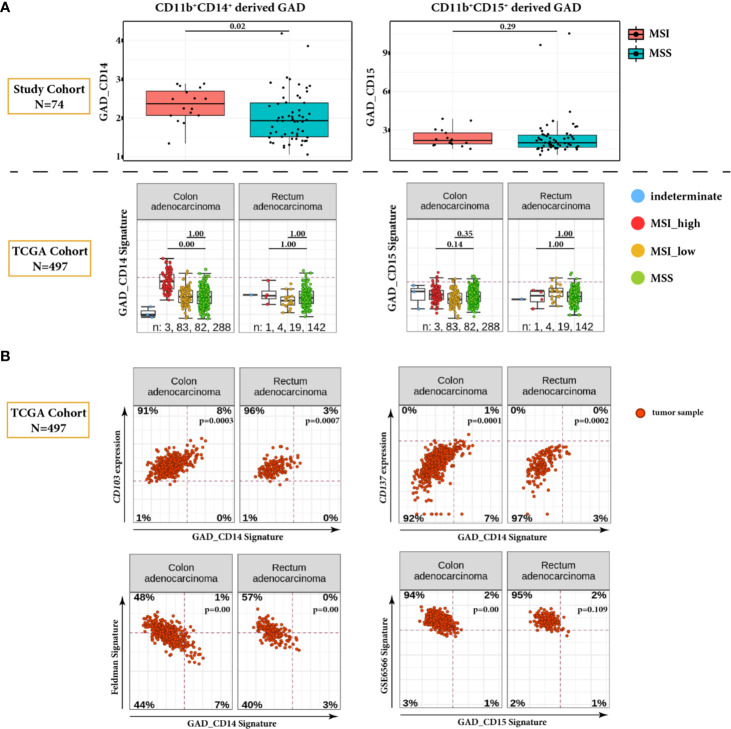
Global Average Distance (GAD) analysis in tumor ROI. **(A)** Comparison of GAD_CD14 and GAD_CD15 parameters between MSI and MSS cases in our study CRC cohort (N = 74) (upper panel). Corresponding GAD derived gene signatures are mapped on the TCGA dataset encompassing 497 CRC tumors, and are presented for MSI_high, MSI_low, MMS, and indeterminate cases (lower panel). For the TCGA dataset, statistical significance is calculated for the differences between MSI_high and MSS categories and between MSI_low and MSS categories. Global average distance between myeloid cells and cytotoxic T cells is significantly higher in MSI cases only for CD11b^+^CD14^+^ cells, which is validated in the TCGA CRC dataset. **(B)** Correlation plots of GAD derived gene signatures and selected genes or gene signatures from TCGA dataset. Positively correlated genes *CD103* and *CD137* and negatively correlated Feldman gene signature are plotted against GAD_CD14. Negatively correlated GSE6566 gene signature is plotted against GAD_CD15. Genes and gene signatures related to anti-tumor specificity, T cell stemness and DC stimulation show association with the Global Average Distance.

In the comparison between MSI and MSS cases, only GAD_CD14 shows significantly higher values in MSI tumors (p = 0.02) (**Figure 3A**). Interestingly, this observation was confirmed when we mapped gene signatures derived from both GAD parameters to the TCGA gene expression data of CRC patients (N = 497) with known MMR status.

Additional analysis of TCGA data set revealed significant negative correlation between GAD_CD14 derived signature and the gene signature representing genes differentiating CD8^+^ TCF7-high (CD8_G, stem-cell-like) vs. CD8^+^ TCF7-low T-cells (CD8_B, exhausted-like or dysfunctional) (Feldman Signature) (17) (**Figure 3B**). Conversely, we found GAD_CD14 derived signature being positively correlated with the expression of *ITGAE* (CD103) and *TNFRSF9* (CD137) genes, which represent activated tumor-specific T-cells (18). GAD_CD15 derived signature shows negative correlation with the GSE6566 signature representing genes differentiating between strongly DC stimulated CD4^+^ T cells (memory cells) vs. weakly DC stimulated CD4^+^ T cells (effector cells) (19).

### Myeloid–T Cell Overlap Allows Grouping Patients According to the Spatial Relation of Immune Suppressive and Effector Cells

**Figure 4:**
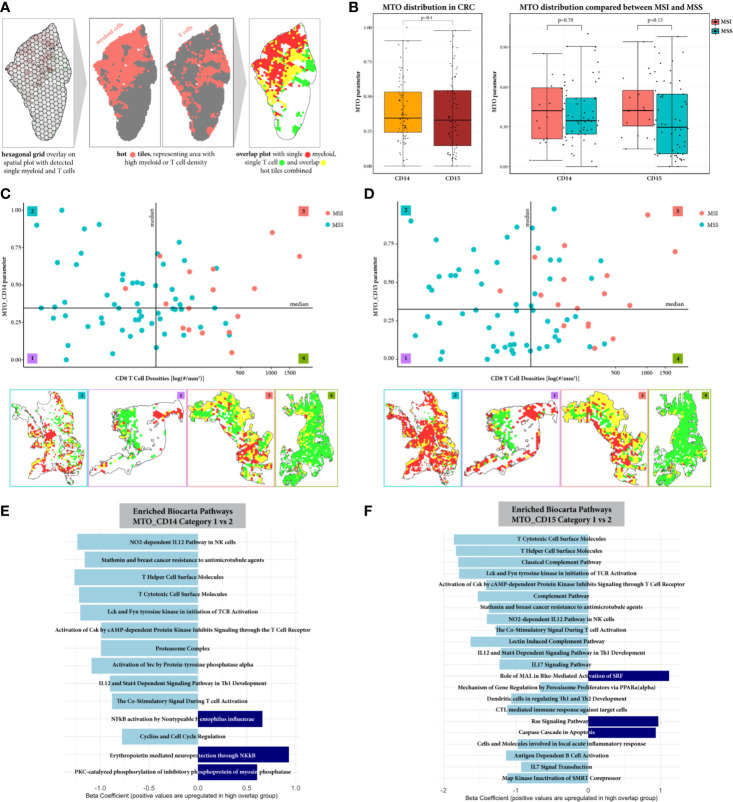
Spatial overlap analysis in tumor ROI. **(A)** Workflow of spatial overlap analysis generating overlap maps on representative example. The tumor annotated sample was overlaid with a hexagonal grid. The density of CD8^+^ T cells and myeloid cells (monocytic or granulocytic, respectively) was identified for every grid tile. A tile was considered as hot for a certain cell type, if its cell density was bigger than the median cell density within the whole cohort. This allowed the visualization of areas showing spatial overlap between T cells and myeloid cells. **(B)** The MTO parameter distribution is represented for both myleoid cell types CD11b^+^CD14^+^ and CD11b^+^CD15^+^, showing an overall similar distribution within the CRC cohort and when comparing between MSI and MSS cases. **(C**, **D)** Stratification of the samples according to CD8^+^T cell density and Myeloid-T cell overlap (MTO). Exemplary overlap plots are depicted for each category: low/low (1), high/low (2), high/high (3) and low/high (4), for MTO_CD14 and MTO_CD15 respectively. Both, MSI and MSS cases are distributed in categories with low and high spatial overlap between myeloid and T cells, suggesting that the MTO analysis adds value to the characterization of CRC patient samples. **(E**, **F)** Pathway Enrichment Analysis represented for comparison between category 1 and 2, respectively. Significantly down-regulated pathways are colored in light blue, significantly up-regulated pathways are colored in dark blue. The comparison between category 4 vs 3 computed no significant differences and is therefore not shown. The spatial proximity of myeloid cells to T cells can be connected with a decreased T cell effector function, when CRC samples show low CD8^+^ T cell density. With a high CD8^+^ T cell density, T cells seem to overcome the immunosuppressive TiME.

Since the GAD parameter reflects only general proximity of myeloid and T cells in the TiME, we applied spatial overlap analysis to detect differences in the local myeloid cell and T cell distribution in the tumor ROI (**Figure 4A**). This resulted in the MTO parameter, with both MTO_CD14 and MTO_CD15 parameters showing a very diverse distribution throughout the whole cohort reflecting the heterogeneous pattern of single and overlap tiles. The median values of MTOrscore CD14 and MTO_CD15 are comparable as they represent similar median overlap with cytotoxic T cells (0.35 and 0.33, p = 0.4, respectively). Further comparison between MSI and MSS cases shows that the median MTO is higher in MSI cases for both monocytic or granulocytic myeloid cell subtypes, however the difference is not statistically significant (**Figure 4B**).

Additional stratification of CRC patients (**Figures 4C, D**) resulted in four categories, each being characterized by two independent variables: amount of cytotoxic T cells in the tumor ROI and their spatial distribution in relation to myeloid cells. For MTO_CD14 stratification, 17, 20, 17, and 19 patients were assigned to categories 1 to 4, respectively. Most of the MSI tumors (15 out of 16, 94%) are in categories 3 and 4 (8 and 7 cases, respectively) with only 1 MSI case being assigned to category 2. For MTO_CD15 stratification, 21, 16, 21, and 16 patients were assigned to the respective categories. Similarly to MTO_CD14 stratification, the majority of MSI cases (16 out of 17 total, 94%) are found in categories 3 and 4 (11 and 5, respectively) with 1 MSI case in category 2. MSS cases are present in all four categories, with categories 1 and 2 almost being exclusive for MSS cases and categories 3 and 4 showing a mix of MSS with MSI cases. The comparison of MTO_CD14 and MTO_CD15 stratification shows that 31% (N = 23) of the CRC patients are differently distributed between MTO low and high irrespective of the CD8^+^ T cell density.

We further investigated differences between CRC samples categorized for low and high spatial overlap of myeloid and T cells using differential gene expression and subsequent pathway enrichment analysis. When comparing CRC tumors with lower CD8^+^ T cell density (categories 1 and 2), samples in category 2 characterized by high MTO show down-regulation of pathways mainly related to T cell differentiation, T cell activation and pro-inflammatory cytokine and chemokine release (**Figures 4E, F**). The MTO_CD15 samples generally reveal more significantly down-regulated pathways than MTO_CD14 samples in category 2, including additional cytokine IL17 and IL7 signaling, complement activation and DC regulation of T helper cells 1 and 2 development pathways. Up-regulated pathways, on the contrary, found in MTO_CD14 category 2 include NF-kB related pathways, while MTO_CD15 category 2 show significance for Ras signaling and caspase cascade in apoptosis. In contrast to that, CRC tumors highly infiltrated by CD8^+^ T cells (categories 3 and 4) exhibit no significant difference in their pathway enrichment, when comparing between samples categorized in low (category 3) and high (category 4) myeloid and T cell overlap (data not shown).

## Discussion

Currently, patient stratification models focus mostly on the amount of tumor infiltrating CD8+ T cells in CRC tumors. However, our data suggests that both the amount of CD8+ T cells and the spatial relationship between myeloid and T cells should be taken into account in CRC tumor immune-based classifications. As there are only a few good examples of prognostic biomarkers in clinical use for stratifying CRC patients, this observation can be of high relevance. In the present study, we focused on analyzing the myeloid cell compartment in CRC primary tumor samples and its spatial relation to CD8^+^ T effector cells. We observed that tumor invasive margin is the tissue ROI in which most of the immune cell types accumulate. Interestingly, when MSI and MSS cases were compared, CD8^+^ T cell densities were significantly higher in MSI cases in all 3 ROIs, i.e. tumor, invasive margin, and normal colon, whereas myeloid cells showed significantly higher accumulation only in tumor ROI. In addition, cytotoxic T cells tend to heavily infiltrate into the tumor epithelial compartment in contrast to myeloid cells which occupy almost exclusively the tumor stromal compartment (**Figures 1**, **2**). Interestingly, the normal colon ROI shows very similar immune cell infiltration patterns compared to tumor and invasive margin ROIs. It can be explained with the fact that the normal tissue represents a very heterogeneic architecture, including mucosa, submucosa, tertiary lymphoid structures (TLS), and muscle or adipose tissue. In addition, part of the invasive margin region reaches into the normal tissue. All these structures show a different immune infiltration, especially the mucosa and TLS show high densities of mainly cytotoxic and regulatory T cells, but also some myeloid cell subtypes. Previous findings of *Galon et al.* confirmed a prognostic value of CD3^+^ and CD8^+^ T cell distribution in tumor and invasive front ROIs in CRC patients’ stage I–III (20). In addition, detailed analysis of consensus molecular subtypes (CMS) showed high CD8^+^ T cell infiltration in CMS groups 1 (MSI-like) and 4 (mesenchymal) with the latter characterized by high myeloid content and the worst prognosis compared to other 3 CMS groups (4). Due to the lack of clinical follow up data in our study cohort, we could not correlate the myeloid cell content with patients’ prognosis. However, hierarchical clustering (**Figure 2E**) according to immune cell densities in all 3 ROIs resulted in 2 main groups—with higher and lower myeloid content. Of these two, only “myeloid low” group exclusively contains MSS cases, whereas “myeloid high” represents both MSI and MSS phenotypes and has no clear molecular characteristics with respect to the CMS classification. In general, our observations based on comparison of myeloid and T cell densities show that myeloid cell concentration in TiME of CRC is not strongly dependent on the molecular phenotypes. Additionally, there is only weak to moderate positive correlation between CD8^+^ T cells and monocytic and granulocytic densities in tumor ROI, suggesting that not the amount of immune cells but rather their distribution plays a more important role in shaping the TiME. Actually, *Si et al.* found that tumor associated neutrophils (TANs) in head and neck cancer execute their immunosuppressive role when they are in close proximity with T cells (21). Activated T cell densities, however, present both weak to moderate positive or negative correlations with neutrophils depending on TANs immunosuppressive or T cell stimulatory functions, respectively. Our IHC methodology does not allow to identify different subsets of TANs or TAMs. Despite that limitation, we could observe high and moderate association between CD11b^+^CD15^+^ and CD11b^+^CD14^+^ cell densities and ARG1^+^ cell densities, respectively (**Figure 2D**). Therefore, we assume that the density correlations observed in our CRC cohort represent mostly correlations with myeloid immunosuppressive subsets.

When we looked closer into the distribution of myeloid and T cells in TiME by measuring the Global Average Distance (GAD), we found that MSI cases have significantly higher GAD between CD8^+^ T cells and monocytic CD11b^+^CD14^+^ myeloid cells. This is not true, however, for granulocytic CD11b^+^CD15^+^ myeloid cells. These findings were confirmed with subsequent mapping of the gene signatures derived from GAD_CD14 and GAD_CD15 to the TCGA dataset, consisting of 497 CRC samples (**Figure 3A**). Our observation can be potentially explained with the higher tendency of cytotoxic T cells to infiltrate and reside in the tumor epithelium in MSI cases (22), resulting in the bigger spatial separation of monocytic myeloid and CD8^+^ T cells. In addition, the GAD_CD14 derived signature shows negative correlation with TCF7 related signature and positive association with expression of *CD137* and *CD103* genes (**Figure 3B**). It indicates that tumors with lower distance between monocytic myeloid and CD8^+^ T cells may have more TCF7 memory stem-like T cells. As described by Held et al., TCF7^+^ cells represent CD8^+^ T cell population residing predominantly in tumor stroma (23). Therefore, our findings suggest that the close proximity between monocytic myeloid and cytotoxic T cells reflects tumor stromal co-localization of CD11b^+^CD14^+^ myeloid cells with CD8^+^TCF7^+^ stroma-residual stem cell-like T cells. On the contrary, tumors with higher monocytic myeloid to T cell distance seem to have more intra-epithelial activated tumor specific CD8^+^ T cells (CD103 ^+^ and CD137^+^) that are spatially separated from stroma-residual myeloid suppressive cells. For CD11b^+^CD15^+^ granulocytic myeloid cells, the distance to CD8^+^ T effector cells does not correlate with MMR status probably due to the fact that this particular spatial parameter reflects different aspect of TiME not related to the microsatellite stability. In fact, tumor associated neutrophils are very heterogeneous cell population with several pro- and anti-tumor functions (24). They can engage with different tumor resident immune cell types performing either stimulatory or inhibitory functions (25). Our findings suggest that tumors with lower distance between granulocytic myeloid cells and cytotoxic T cells may have higher CD4^+^ memory T cell content. This observation may indicate substituting immunostimulatory role of CD4^+^ memory T cells in the situation when CD8^+^ T cell activity is downregulated by granulocytic immunosuppressive myeloid cells.

Since GAD does not give a detailed insight into the distribution patterns of myeloid and T cells in TiME, we introduced the spatial overlap analysis using the Myeloid T cell overlap (MTO) parameter. Using both the amount of myeloid-T cell overlap and the CD8^+^ T cell density, we assigned CRC patient samples into 1 of 4 categories (low/low, high/low, high/high, or low/high) (**Figures 4C, D**). We observed CRC tumors showing high spatial overlap, with low (category 2, high/low) and high CD8^+^ T cell density (category 3, high/high), reflecting co-localization of the majority of infiltrating T cells with myeloid cells. In comparison, categories containing CRC tumors that show low MTO and either low (category 1, low/low) or high CD8^+^ T cell density (category 4, low/high) represent tumors with low level of co-localization. Interestingly, the MSI cases, which show predominantly high CD8^+^ T cell density, intermingle with MSS cases and are distributed between categories 3 and 4. It potentially indicates that the spatial organization of CRC TiME does not depend on the tumor MMR status but is rather a result of local interactions between myeloid and T cell populations. This is probably the reason why 31% of CRC cases in our cohort are assigned to different overlap-derived categories when MTO_CD14 and MTO_CD15 are compared.

To better reflect the T cell activity in cases showing high amount of spatial overlap areas between immunosuppressive myeloid cells and cytotoxic T cells in tumor ROI, MTO categories 1 (low/low) and 2 (high/low) and categories 3 (high/high) and 4 (low/high) were compared by using differential gene expression and subsequent pathway enrichment analysis (**Figures 4E, F**). Only categories with low CD8^+^ T cell density appear to show significant differences in the regulation of cytotoxic T cell activity. For both MTO_CD14 and MTO_CD15 the category 2 characteristic for high spatial overlap depicts a general down-regulation of T cell related pathways. First, the analysis suggests an impaired IL12 mediated T cell differentiation into T helper 1 (Th1) and Th2 cells when immunosuppressive myeloid cells occupy T cell infiltrated areas in the tumor ROI. This is followed by a reduced cytotoxic T cell activity, marked by lower expression of cell surface molecules, a dysfunctional activation initiation of the T cell receptor (TCR) and by down-regulated cytokine signaling. These findings are in line in with the described in literature mechanisms of T cell suppression by activated neutrophils requiring direct contact between them and T cells (26). While the effects on cytotoxic T cell function is very similar, functional differences between monocytic and granulocytic myeloid cells are mainly detected in pathways up-regulated for NF-kB signaling (CD11b^+^CD14^+^) and Ras signaling and apoptosis (CD11b^+^CD15^+^). It may indicate differences in cell specific functions, e.g. changes in monocytic MDSCs pro-inflammatory function and anti-tumor activity of neutrophils (27, 28). On the contrary, the comparison between MTO categories characteristic for high CD8^+^ T cell density revealed no significant differences in the functional status of cytotoxic T cells. These results suggest that with increased infiltration by CD8^+^ T cells the local immunosuppressive effect of interacting myeloid cells is overcome or is not dominating any longer in TiME.

Our study has certain limitations. The CRC cohort we analyze is relatively small (N = 74) and misses clinical follow-up information. We partially address it by validating our results on the TCGA database through mapping of the distance derived signatures. In addition, we used CMS classification as a surrogate of the clinical outcome. Due to the limitation of IHC methodology and bulk gene expression analysis using whole tissue sections we could not study presence and location of other types of cells (e.g. cancer cells, fibroblasts, certain immune cell subsets) that may have potential impact on the distribution of T cells. One of the solutions to that problem could be application of multiplex immunofluorescence and spatial genomics methods which would allow detailed analysis of several tumor compartments and more complex immune cell phenotypes. Instead, we used image registration capabilities of HALO software to analyze several immune cell types in the same coordinate system.

In summary, this study presents a multimodal approach addressing the distribution of myeloid and T cells in the TiME of CRC tumors. We combine digital image-based analysis, including cell density, cell-to-cell distance and spatial overlap, with gene expression profiling to link the tumor spatial features with the biological function of tumor infiltrating immune cells. Importantly, our data shows that myeloid cells, in general, play a crucial role in building the TiME of CRC tumors. In our cohort, we observe high variability of tumor infiltration pattern by monocytic and granulocytic myeloid cells and their spatial relation to cytotoxic T cells. Our findings suggest that the location and the function of CD8^+^ T effector cells is influenced by the tumor stroma-residual myeloid cells. In particular, GAD_CD14 derived gene signature indicates that the location of monocytic cells correlates with the distribution of TCF7 memory stem-like lymphocytes and tumor specific T cells. Additionally, the spatial overlap analysis shows the suppressive functional effect of both monocytic and granulocytic myeloid cells on cytotoxic T cells, when co-localizing in immune dense areas in the tumor ROI. Given that current patients stratification models are focusing mostly on the amount of tumor infiltrating CD8^+^ T cells, results of our study provide strong rationale for including spatial relation between myeloid and T cells into CRC tumor immune-based classifications. Our system for characterization of CRC samples, based on both spatial relationship and T cell density, is a promising tool for investigation as a potential prognostic biomarker for CRC and warrants additional investigation. Further validation is needed to correlate this tool with clinical outcome in the hope of supporting patients’ enrichment strategies.

## Data Availability Statement

The datasets presented in this study can be found in online repositories (https://www.ncbi.nlm.nih.gov/geo/query/acc.cgi?acc=GSE152395).

## Ethics Statement

Human primary CRC tumor specimens of 74 treatment-naïve patients were acquired from Avaden Biosciences and Indivumed. The samples were collected after obtaining patients informed consent and approval from the respective Institutional Review Boards or equivalent agencies.

## Author Contributions

NZ, MC, FG, and KK contributed conception and design of the study. NZ organized tissue samples and data base, performed all IHC stainings, tissue annotations, and digital image analysis. KK provided quality control for annotations of digital images of histopathological slides. HF performed spatial statistics, CH-O performed gene expression and correlative analysis with distance parameter. DPH performed differential gene expression and pathway analysis. NZ and KK wrote the manuscript. All authors contributed to the article and approved the submitted version.

## Funding

The whole study has been sponsored by Roche through the internal PhD program. The funder was not involved in the study design, collection, analysis, interpretation of data, the writing of this article or the decision to submit it for publication.

## Conflict of Interest

All the authors are employees of Roche. DH was employed by Genentech, Inc.
