# Supplementary material for: Analysis of Spatial Organization of Suppressive Myeloid Cells and Effector T Cells in Colorectal Cancer—A Potential Tool for Discovering Prognostic Biomarkers in Clinical Research
Source: Front Immunol. 2020 Oct 29;11:550250. doi: 10.3389/fimmu.2020.550250 (PMC7658632; doi:10.3389/fimmu.2020.550250)
Supplement: Supplementary file 1 [file DataSheet_1.pdf]

# Supplementary Material

## 1 SUPPLEMENTARY DATA

Supplementary Material should be uploaded separately on submission. Please include any supplementary data, figures and/or tables. All supplementary files are deposited to FigShare for permanent storage and receive a DOI.

Supplementary material is not typeset so please ensure that all information is clearly presented, the appropriate caption is included in the file and not in the manuscript, and that the style conforms to the rest of the article. To avoid discrepancies between the published article and the supplementary material, please do not add the title, author list, affiliations or correspondence in the supplementary files.

## 2 SUPPLEMENTARY TABLES AND FIGURES

For more information on Supplementary Material and for details on the different file types accepted, please see the Supplementary Material section of the Author Guidelines.

Figures, tables, and images will be published under a Creative Commons CC-BY licence and permission must be obtained for use of copyrighted material from other sources (including re-published/adapted/modified/partial figures and images from the internet). It is the responsibility of the authors to acquire the licenses, to follow any citation instructions requested by third-party rights holders, and cover any supplementary charges.

### 2.1 Figures

**Figure S1.** Enter the caption for your figure here. Repeat as necessary for each of your figures

A

B

**Figure S2.** This is a figure with sub figures, **(A)** is one logo, **(B)** is a different logo.

### 2.2 tables

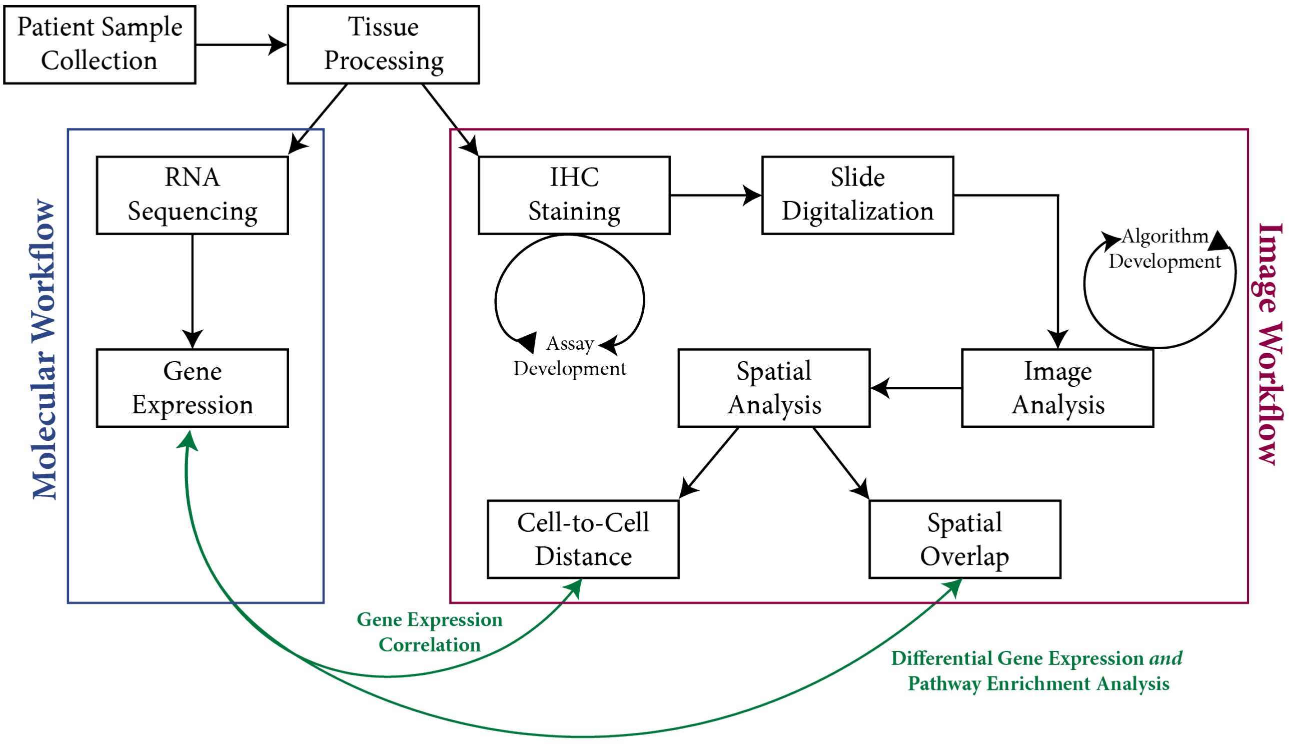


**Figure S3.** Proposed Study Workflow. This figure represents the schematics of the tissue analysis workflow used in the presented study. Image Workflow (red box) shows all the steps, starting from IHC assay development through slide digitalization up to spatial image analysis. This combined with the Molecular Workflow (blue box), including RNA Sequencing and Gene Expression Profiling.

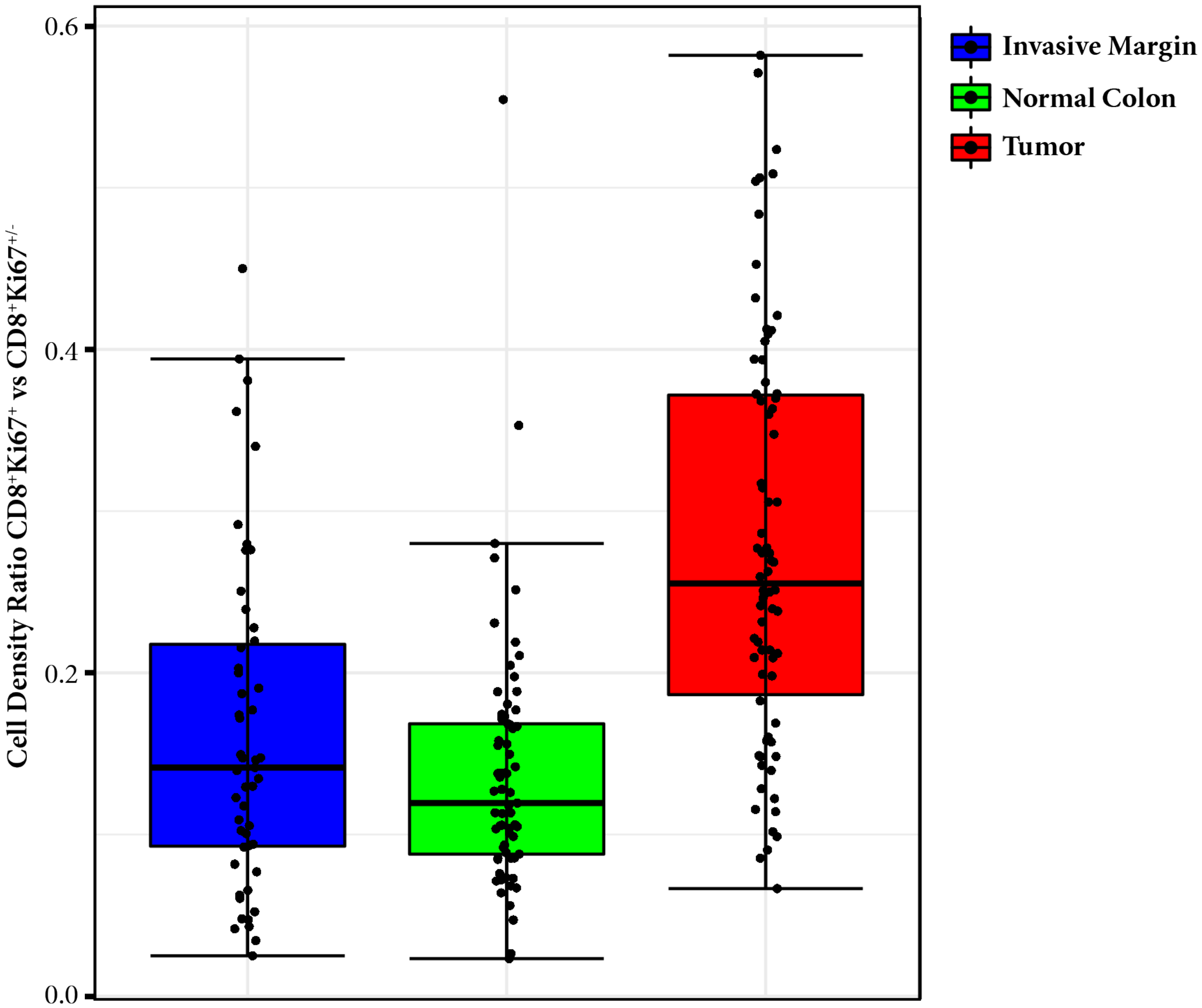


**Figure S4.** Ratio between $CD8^{+}Ki67^{+}$ and $CD8^{+}Ki67^{+/-}$ T cell densities. The proportion of proliferating cytotoxic T cells is presented for all three ROIs, including invasive margin, normal colon and tumor. Generally, the highest proliferation activity of $CD8^{+}$ T cells is detected within tumor ROI.

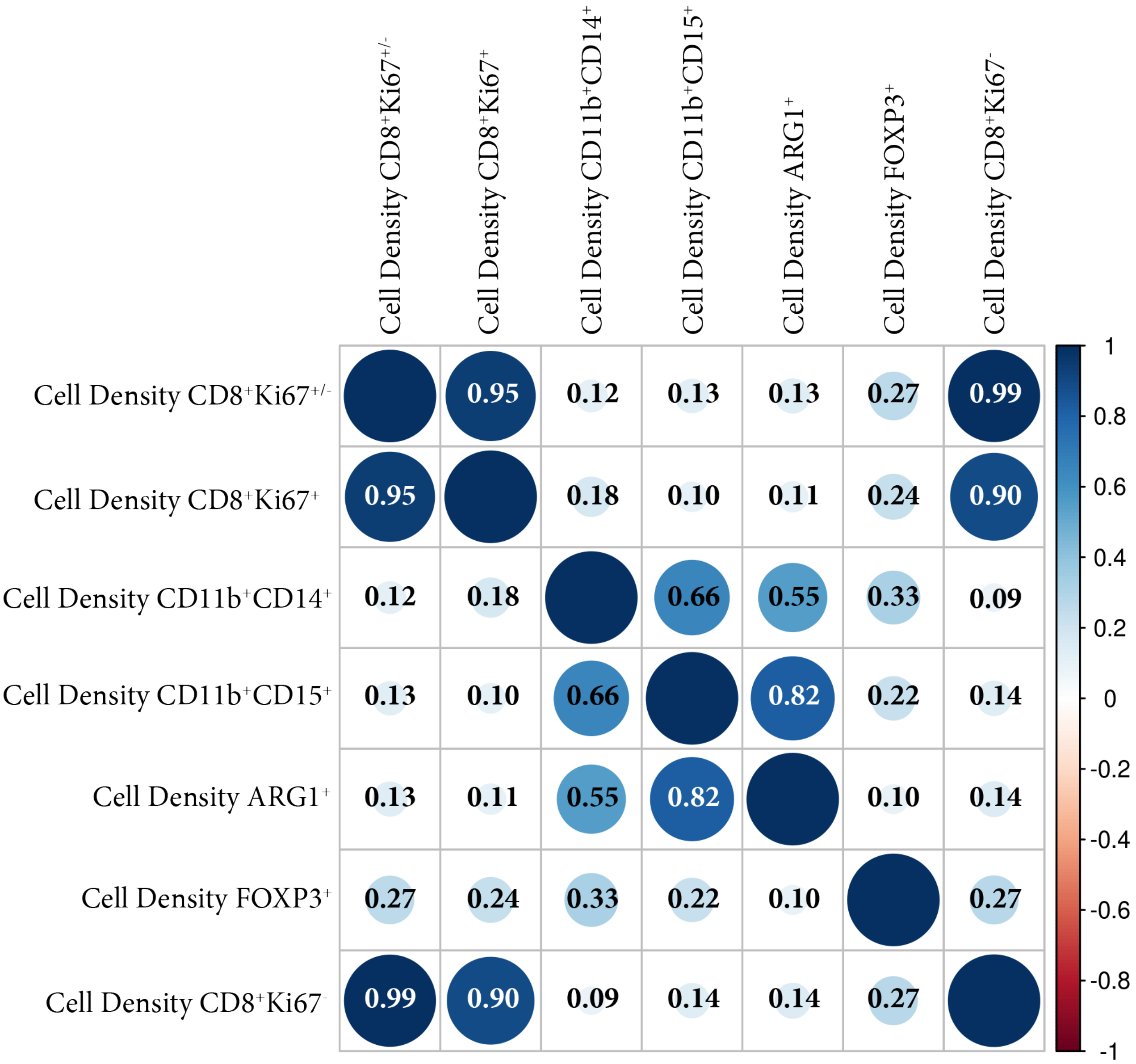


**Figure S5.** Cell Density Correlation Analysis in the invasive margin ROI. The circle size corresponds to the strength of the Pearson correlation coefficient and the color corresponds to either positive (blue) or negative (red) correlation. The correlation between cell densities is comparable to computed correlations in the tumor ROI.

**Table S1.** Technical abbreviations and short descriptions.

| Abbreviation | Parameter | Description |
|---|---|---|
| CIT | Cancer Immunotherapy | Type of cancer treatment with immunomodulating medicines. |
| CMS | Consensus Molecular Subtypes | Colorectal Cancer subtyping according to gene expression profiling. |
| CRC | Colorectal Cancer | Tumor indication. |
| FFPET | Formalin fixed and paraffin embedded tissue | Processed tumor specimens in the form of a paraffin block. |
| GAD | Global Average Distance | Non-normalized parameter characterizing the average distance between myeloid and T cells computed in the tumor region of interest of each colorectal cancer sample. |
| $GAD_{norm}$ | Normalized Global Average Distance | GAD parameter normalized against the random distribution and the density of myeloid cells in the tumor area. |
| GAD_CD14 | Normalized GAD for $CD11b^+CD14^+$ myeloid cells | Normalized parameter characterizing the average distance between monocytic myeloid and T cells computed in the tumor region of interest of each Colorectal Cancer sample. |
| GAD_CD15 | Normalized GAD for $CD11b^+CD15^+$ myeloid cells | Normalized parameter characterizing the average distance between granulocytic myeloid and T cells computed in the tumor region of interest of each Colorectal Cancer sample. |
| IHC | Immunohistochemistry | Antibody-based method of detecting protein antigens in a fixed tissue. |
| MDSC | Myeloid-Derived Suppressor Cells | Immature myeloid cells with immunosuppressive function. |
| MMR | Mismatch Repair Status | Tumor subtyping according to the mutational status of the mismatch repair proteins. |
| MSI | Microsatellite Instability | Deficiency in mismatch repair machinery. |
| MSS | Microsatellite Stability | Proficiency in mismatch repair machinery. |
| MTO | Myeloid-T cell Overlap | Parameter representing the tiles infiltrated by myeloid and T cells (spatial overlap), normalized against the total number of tiles infiltrated by T cells. |
| MTO_CD14 | Myeloid-T cell Overlap for $CD11b^+CD14^+$ myeloid cells | Parameter representing the tiles infiltrated by monocytic myeloid and T cells (spatial overlap), normalized against the total number of tiles infiltrated by T cells. |
| MTO_CD15 | Myeloid-T cell Overlap for $CD11b^+CD15^+$ myeloid cells | Parameter representing the tiles infiltrated by granulocytic myeloid and T cells (spatial overlap), normalized against the total number of tiles infiltrated by T cells. |
| ROI | Region of Interest | Annotated areas in the slide images used for density and spatial analysis. |
| TCGA | The Cancer Genome Atlas | Public repository of broad of spectrum of tumors, including clinical and molecular information. |
| TiME | Tumor Immune Microenvironment | The immune components of the tumor milieu. |
| TIL | Tumor-infiltrating lymphocytes | T lymphocytes infiltrating the tumor bed. |
| TLS | Tertiary Lymphoid Structure | Immune cell aggregates having a certain composition and architectural pattern. |
| TMB | Tumor Mutational Burden | Number of mutations per base pair. |

**Table S2.** Genes positively and negatively correlating with distance derived GAD parameters. Listed genes represent the respective surrogate gene signatures for GAD_14 and GAD_CD15, used for mapping to TCGA dataset. The cutoffs after gene expression correlation with continuous values were set at $|R| > 0.4$ for GAD_14 and at $|R| > 0.3$ for GAD_CD15.

| Distance Parameter | Genes |
|---|---|
| **GAD_CD14** | ATF5, TNFRSF9, CRTC3, CHST12, FCHSD2, FOXP3, PRPF31, USF2, GYPC, TTBK1, CXCL13, CD2, SCPEP1, ALKBH5, ZNF831, SENP3, LLGL1, CST7, CALM3, CASS4, PNMA3, ARSF, RTN1, C18orf25, MTMR9LP, KLHL22, RNF34, FAM26F, METRNL, ITGAE, TANGO2, ICAM5, -FEZF1, -BHMT, -MTRNR2L1, -RANBP6, -NOTUM, -ZP3, -PCLO, -SLC26A8, -CASC9, -KCTD4, -SLC35D2, -DRD1, -KRT39, -PGAP3, -KLHL9, -C5orf63, -TTC30A, -MIR374C, -ETV4, -SPATA24, -GABRB1, -WNK4, -TRIM67, -ALAS2, -SOX14, -ERV3-1, -SHANK2-AS3, -TM4SF20, -RAB3GAP2, -DSG1, -SPATA6L, -TAF7 |
| **GAD_CD15** | -ITPKB-IT1, -EPN3, -PABPC4, -ABCC12, -KPNA4, -CKB, -BANF2, -PRDM14, -XKR3, -VPS28, -SLC10A5, -TOB1, -HOXC11, -ITLN1, -CEACAM22P, -SHARPIN, -ZFAND1, -OR6B1, -NMU, -CREG2, -LRP2,-MIR6837, -FAM13A, -UGT1A4, -UQCRB, -IGFBP2, -OBFC1, -FA2H, -RAB3GAP2, -LINC00622, -YOD1, -OR5AK2, -KRT1, -OCIAD2, -C16orf80, -CA12, -CANT1, -B4GALNT2, -HERC2P10, -ATAD1, -EFHB, -CCDC47, -PPP1R15B, -MIR6887, -SPATC1L, -SLC26A4-AS1, -TMEM105, -CA5A, -DRP2, -ARL14, -SMG1P5, -TMEM68, -KRT80, -MTSS1L, -GSTO2, -UGT3A1, -YWHAQ, -PRKAG3, -C4orf36, -ZDHHC11, -CCDC42B, -CXADRP3, -NT5C2, -EGR4, -GYS2, -FAM84B, -GOLT1A, -AGPAT9, -SPRED2, -PWWP2B, -LINC00521, -CSN1S2AP, -LYPLA1, -CEMIP, -FRAS1, -ANKRD30B, -ANKRD22, -KRT23, -COX6C, -PCOLCE2, -TEX26, -PHGDH, -PKHD1, -TCEA1, -CCER2, -ULBP1, -COMMD2, -PDCL2, -BEGAIN, -KCNJ16, -LGALS12, C2, ICAM5, OR2T5, FAM231A, GLCCI1, MSL3, KIAA1328, DCC, ZBTB40, KCTD20, HAND2-AS1, FAM181A, ZNF271, RHOXF1, RNF216P1, TFPT, CHD5, PURA, CNTF, DNAJB6, TECTA, CXorf40A, OR11H12, PLXNA3, HTR7, MIR4644, C18orf25, ZNF175, RCN3, CBL, UBR2, UBE4A, ZNF543, CD99L2, NUBP1, RRAGB, SYT15, CYB5R4, PLEKHA8, C14orf183, EGFL8, FAXDC2, STAG3L4, LMOD2, PSMC2, POLI, MIR4744, PIK3C3, C2orf71, KMT2A, A1BG, MIR135A1, ZNF18, FAM21A, ERMARD, ZNF101, SEPT1, ZCCHC5, PPP2R2B, FKBP14, PQLC3, TBX19, SNORD11B, ZNF324, HAVCR1P1, NRG4, IFNG, ANKRD34B, TRNT1, C1QTNF9, ATP11C, ZNF547, SLC10A4, CD19, SIT1, RARS, OGDH, ABCD1, SLFNL1-AS1, PPP1R14A, GOLGA6L6, SORCS2, LINC00506, IRX6, DPF2, PSORS1C3, MIR3183, TRA2A, NEFH, YPEL2, PACS1, RPA3OS, GOLGA6L1, RNF216, OR2T29, CNPY3, NAPA-AS1, NUPL2, MIR3939, MIR218-1, TCP11L2, ELP2, MIR5739, RAP2C, S100B, ARSF, FOXE1, LINC00630, C10orf107, IMPG2, INCA1, METTL16, CALCOCO1, UBASH3A, TBC1D22A, PMM2, ZNF583, RPL36A-HNRNPH2, MICA, ZNF354A, SNRNP70, ANKRD44-IT1, SCML4, SPACA3, IL6, XPC, C4B, PNRC1, C20orf166, HAUS1, TBRG1, SUMF2, MIR3683, OR11H2, KIAA1024, FBLN7, SLC8A2, ZNF783, LSMEM1, LIME1, VAMP2, TIRAP, METTL25, MIR381, CUL9, MIR450A1, KBTBD8, FOXO3, ANKRD49, ACSM5, EVL, TMEFF2, APBB1, MIR197, GNA14-AS1, GYPC, MAGOH2, TSPY1, CHST12, RNASEK, FAM226A, FAM226B, APBB3, PRPF31, ZNF79, RANBP17, ACTL8, ZC2HC1B, MIR5582, IRAK4, POLN, SLC6A7, MIR181A1HG, GHRL, CD160, FUNDC2P2, SNORD109B, ZNF655, ITPR1, TBP |
